# Detecting Disruption of HER2 Membrane Protein Organization in Cell Membranes with Nanoscale Precision

**DOI:** 10.1021/acssensors.3c01437

**Published:** 2023-11-13

**Authors:** Yasaman Moradi, Jerry S. H. Lee, Andrea M. Armani

**Affiliations:** † Mork Family Department of Chemical Engineering and Materials Science, University of Southern California, Los Angeles, California 90089, United States; ‡ Ellison Institute of Technology, Los Angeles, California 90064, United States; § Keck School of Medicine, University of Southern California, Los Angeles, California 90089, United States

**Keywords:** aggregation-induced emission, fluorescent sensor, imaging, microscopy, transmembrane proteins

## Abstract

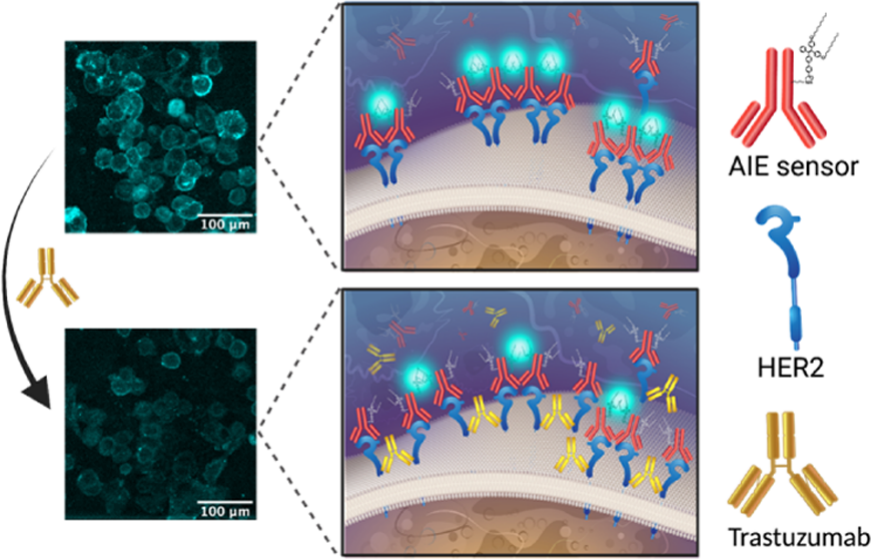

The spatiotemporal organization of proteins within the cell membrane can affect numerous biological functions, including cell signaling, communication, and transportation. Deviations from normal spatial arrangements have been observed in various diseases, and a better understanding of this process is a key stepping stone to advancing development of clinical interventions. However, given the nanometer length scales involved, detecting these subtle changes has primarily relied on complex super-resolution and single-molecule imaging methods. In this work, we demonstrate an alternative fluorescent imaging strategy for detecting protein organization based on a material that exhibits a unique photophysical behavior known as aggregation-induced emission (AIE). Organic AIE molecules have an increase in emission signal when they are in close proximity, and the molecular motion is restricted. This property simultaneously addresses the high background noise and low detection signal that limit conventional widefield fluorescent imaging. To demonstrate the potential of this approach, the fluorescent molecule sensor is conjugated to a human epidermal growth factor receptor 2 (HER2)-specific antibody and used to investigate the spatiotemporal behavior of HER2 clustering in the membrane of HER2-overexpressing breast cancer cells. Notably, the disruption of HER2 clusters in response to an FDA-approved monoclonal antibody therapeutic (Trastuzumab) is successfully detected using a simple widefield fluorescent microscope. While the sensor demonstrated here is optimized for sensing HER2 clustering, it is an easily adaptable platform. Moreover, given the compatibility with widefield imaging, the system has the potential to be used with high-throughput imaging techniques, accelerating investigations into membrane protein spatiotemporal organization.

Transmembrane proteins play a critical role in governing fundamental cell processes, such as cell signaling and cell division. However, in many cases, it is not simply the presence or absence of a given protein but its spatiotemporal organization within the membrane that modulates biological processes.^1,2^ Therefore, the ability to decipher these dynamic interactions is key to unraveling how they mediate the cellular signal transductions, which play a role in a range of health conditions.

At a fundamental science level, several methods exist for measuring the spatial organization of membrane receptors, including electron microscopy,^3,4^ optical and fluorescence microscopy-based techniques,^5−10^ and proximity-based assays.^11−13^ Among these, electron microscopy provides the highest resolution. However, because it is not compatible with live cells, it can only provide static snapshots of dynamic processes that do not fully capture the behavior of receptors in the membrane. Alternatively, super-resolution imaging technologies can be used. However, these are not conducive to high-throughput sample analysis methods and rely on extremely specialized instrumentation.^14^ Given the complexity of these interactions, the ability to acquire large data sets and perform a high-dimensional analysis is key to unraveling the underlying biological control mechanisms.

To overcome these challenges requires rethinking our approach to imaging. One strategy is to develop new types of fluorescent probes that can serve as proximity sensors and operate in a high-throughput system, and one promising material system is based on organic molecules that exhibit aggregation-induced emission (AIE) behavior.

Aggregation-induced emission (AIE) describes a photophysical property in which the fluorescent intensity of a fluorophore aggregate is higher than when the molecule is well dispersed in solution.^15^ This behavior is attributed to a restriction of the intramolecular rotations, which increases the emissivity of the molecule.^16,17^ In contrast, most commercially available fluorescent compounds exhibit the opposite behavior due to strong π–π interactions, and their emission is reduced or completely quenched when they aggregate in the solid state or are in high-concentration solutions.^17,18^ To date, AIE molecules have been used for biomolecule sensing in solution,^19−22^ bioimaging,^23−28^ monitoring protein folding/unfolding processes in cells,^29^ and sensing the interaction of proteins in solution.^30−32^ Therefore, a material exhibiting an AIE response has the potential of being utilized for studying the spatial arrangement of membrane receptors on the nanometer length scale using conventional fluorescent microscopy.

In this work, we develop a fluorophore based on tetraphenylethylene (TPE), an AIE molecule, for studying the clustering behavior of HER2 in HER2-overexpressing breast cancer cells and investigate the response of the HER2 cluster to a therapeutic. HER2 is involved in the regulation of several signaling pathways that lead to an increase in cell proliferation in several types of cancer.^33,34^ Because HER2 is known to localize in clusters on the membrane,^3,35,36^ one therapeutic strategy is the disruption of this organization.^37,38^ To study this dynamic nanoscale process, the TPE-based probe is conjugated to an antibody specific to the extracellular domain of HER2. As shown in Figure 1a, when the HER2 proteins are separated, the developed fluorophore sensor is not emissive. However, when the concentration of HER2 increases and clusters form, the molecule undergoes a fluorescent turn-on process due to the AIE behavior of the TPE (Figure 1b). Because only HER2 molecules in close proximity initiate the turn-on process, this approach overcomes many of the previous limitations by providing a method to detect HER2 clustering and simultaneously reducing background noise. Furthermore, since AIE is a reversible process, the dynamic interactions of HER2 proteins in response to external stimuli, such as therapeutics, can be studied.

**Figure 1:**
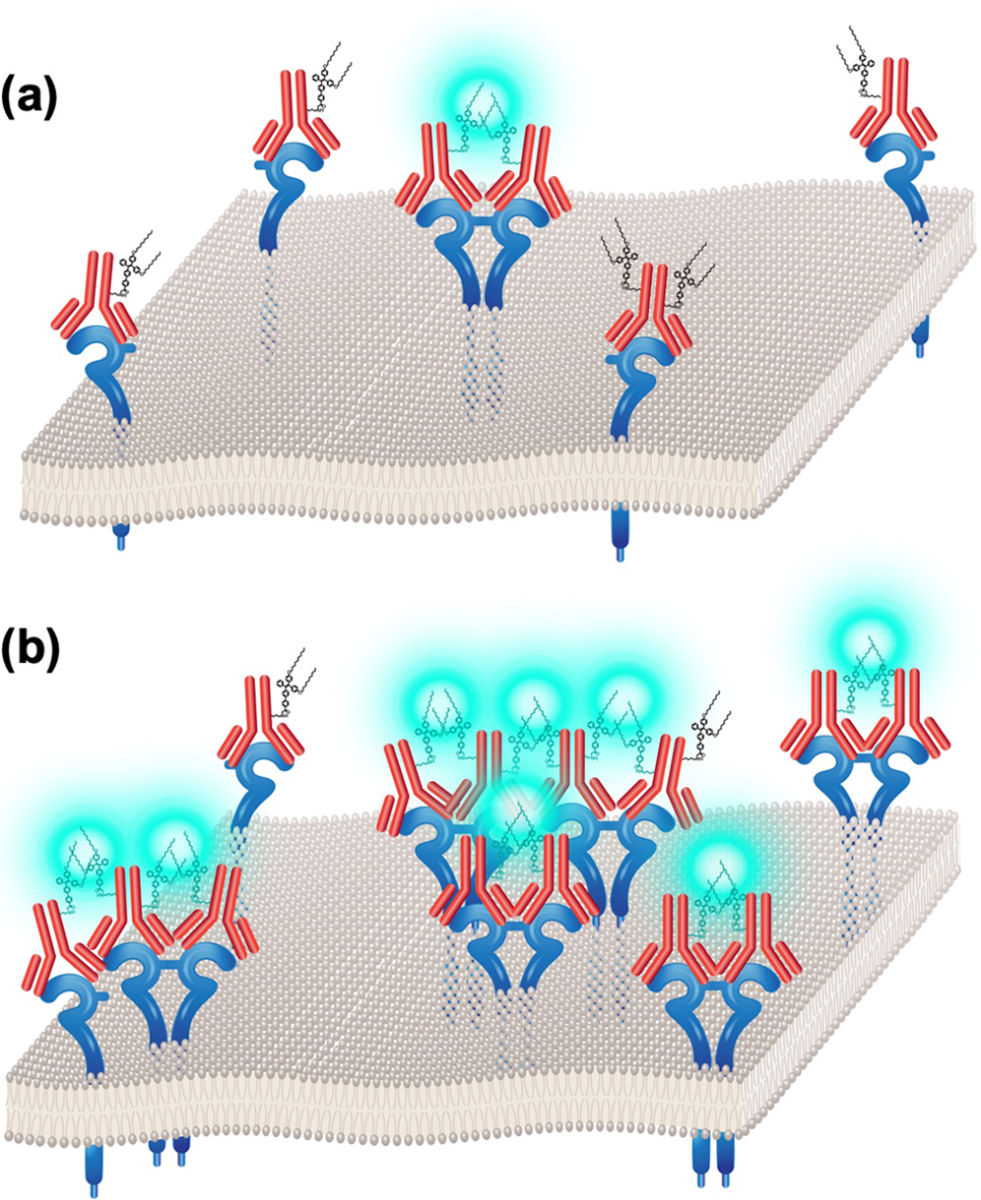
Schematic of AIE sensor for localized detection and visualization of HER2 clusters or HER2s that are localized in each other’s proximity on (a) HER2-negative (low HER2 expression) and (b) HER2-positive (overexpressing) cell surfaces.

## Experimental Section

The detailed information on the chemicals and reagents, instrumentation, synthesis, bioconjugation, and characterization results of the intermediates and final molecule as well as the validation of the cell lines are presented in the Supporting Information.

### Synthesis and Characterization of the AIE Sensor

The synthetic details and NMR spectra confirming the synthesis of TPE-NHS are included in the SI. Post synthesis, the absorption and emission spectra of TPE-NHS were characterized in distilled water, DMSO, and DMEM cell media. Furthermore, the AIE response of TPE-NHS molecule was confirmed. Lastly, the potential for Trastuzumab to directly interact with TPE-NHS was studied. All details and results are listed in SI.

To develop the AIE sensor, the TPE-NHS molecule was conjugated to HER2-specific antibody through a micelle-mediated bioconjugation reaction that leveraged the intrinsic lysine residues of the HER2-specific antibody. The details of the bioconjugation reaction, purification of free TPE-NHS using a gel spin column, conjugation confirmation, and optical characterization methods are included in the SI.

### Direct Immunofluorescent Imaging

SKBR3 cells (ATCC, HTB-30) and MCF7 cells (ATCC, HTB-22) were seeded at the density of 7,000 cells per well in a 96-well glass-bottom plate (Cellvis, P96-0-N) and incubated for 3 days before running the assay to reach the approximate confluency of 70%. After 3 days, the medium was removed, and the cells were fixed using 4% Paraformaldehyde (Alfa Aesar, J62478), washed 3 times (5 min each), and blocked using 2% BSA blocking buffer (Thermo Scientific 37525) for 1 h. The antibody dye conjugates (Fluorescein-HER2 Ab as the positive control and different TPE-HER2 Ab conjugates) were diluted to the concentration of 10 μg/mL in 0.1% BSA solution, added to the fixed SKBR3 and MCF7 cells, and left at 4 °C overnight. The samples were washed with 1× PBS 2 times and imaged on a Zeiss Axio Observer connected to an X-cite Series 120Q light source using the excitation and emission filters of interest using a 20× objective. The TPE filter cube has an excitation of G365, BS of 395, and emission BP of 535/30. The Fluorescein filter cube has an excitation BP of 500/25, BS of 515, and emission BP of 535/30.

### Colocalization Immunofluorescent Imaging

SKBR3 cells were seeded at the density of 7,000 cells per well in a 96-well glass-bottom plate (Cellvis, P96-0-N) and incubated for 3 days before running the assay to reach the approximate confluency of 70%. After 3 days, the media was removed, and the cells were fixed using 4% paraformaldehyde (Alfa Aesar, J62478), washed 3 times (5 min each), and blocked using 2% BSA blocking buffer (Thermo Scientific 37525) for 1 h. Then, a staining solution consisting of 20 μg/mL TPE-HER2 Ab and 20 μg/mL Texas Red-HER2 Ab (Invitrogen, T20175) in 0.1% BSA was added to the cells. After overnight staining at 4 °C, fixed SKBR3 cells were washed and imaged in the bright-field channel, TPE channel (filter cube with the excitation of G365, BS of 395, and emission BP of 535/30), and Texas Red channel (filter cube with the excitation BP of 550/25, BS of 570, and emission BP of 605/70) on Zeiss Axio Observer widefield fluorescent microscope using a 20× objective.

### Analysis of the Impact of Trastuzumab on HER2 Clusters

SKBR3 cells (ATCC, HTB-30) were seeded at the density of 7000 cells per well in a 96-well glass-bottom plate (Cellvis, P96-0-N) and incubated for 3 days. After reaching the ideal cell confluency on day 3, different concentrations of Trastuzumab (Selleckchem, A2007) in McCoy media (from 0 to 100 μg/mL) were prepared by serial dilution. The old cell media was replaced with the Trastuzumab media and incubated for 2, 8, and 24 h. Then, cells were fixed and washed by following the direct immunofluorescent protocol. TPE-HER2 Ab and Fluorescein-HER2 Ab at concentrations of 10 μg/mL in 0.1% BSA solution were added to the fixed cells and left at 4 °C overnight. The samples were washed with 1× PBS 2 times and imaged on a Zeiss Axio Observer using a 20× objective. The TPE filter cube with excitation of G365, BS of 395, and emission BP of 535/30 and the Fluorescein filter cube with excitation BP of 500/25, BS of 515, and emission BP of 535/30 were used. The imaging parameters were held constant for all of the wells, allowing this value to be used for comparison across conditions. Postimaging, the data was analyzed based on the description in the Image Analysis section.

### Image Analysis

An in-house developed image analysis tool that is available on GitHub was used to track the fluctuations in the fluorescent signal post treatment. The image analysis tool uses an edge detection algorithm to define the region of interest (ROI), which is the cell area. Then, the defined mask was applied to the fluorescent image. Summation of the pixel values of the masked region and the total masked area (number of pixels) was extracted from each fluorescent image. Dividing the summation of the pixel values by the masked area, a value representing the average fluorescent intensity per fluorescent image was calculated. A detailed description of the image analysis platform is included in the SI.

## Results and Discussion

### Design Rationale of the AIE Molecule

The primary design criteria for the fluorescent sensor are to maintain high biological specificity while achieving nanoscale spatial sensitivity. One common strategy to endow specificity to a fluorescent probe is to conjugate the molecule directly to the amine groups that are located on the Fc-region of the antibody.^39^ In this work, the TPE-based molecule was functionalized with *N*-hydroxy succinimide (NHS) ester, which subsequently bound to a monoclonal antibody specific to HER2.^39,40^ In addition, a pair of hydrocarbon chains were added to increase the probability of intermolecular restriction as the HER2 proteins cluster.

**Figure 2:**
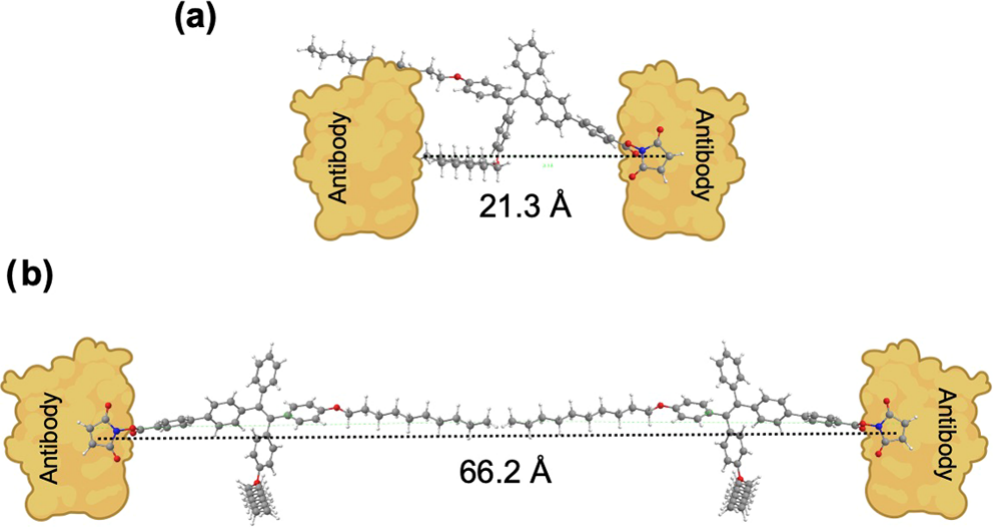
Distance of the NHS ester portion of TPE-NHS from the end of the hydrocarbon chains in the ground state can determine the detection range of the AIE sensor. The TPE-bound antibodies binding to HER2 protein can detect (a) a minimum distance of 21.3 Å upon interaction of the TPE-NHS molecule from the shorter side with the surface of a nearby HER2-bound antibody and (b) the maximum distance of 66.2 Å upon interaction of two molecules from their longest length.

The length of the TPE-NHS was modeled using density functional theory (QChem). This value sets the minimum and maximum interaction distances between a pair of TPE molecules that could give rise to the AIE response. Based on the results, detection of TPE-NHS is in the range of 2–6 nm (Figure 2). Additional details on the modeling are presented in the SI (Figure S1).

### Synthesis and Characterization of the AIE Molecule

The synthesis process of TPE-NHS molecule is shown in Scheme 1a,^41−43^ and the different chemical groups of TPE-NHS are color-coded to highlight their role in the molecule’s operation. The intermediate and final structures were confirmed with NMR (Figures S2–S9).

**Scheme 1:**
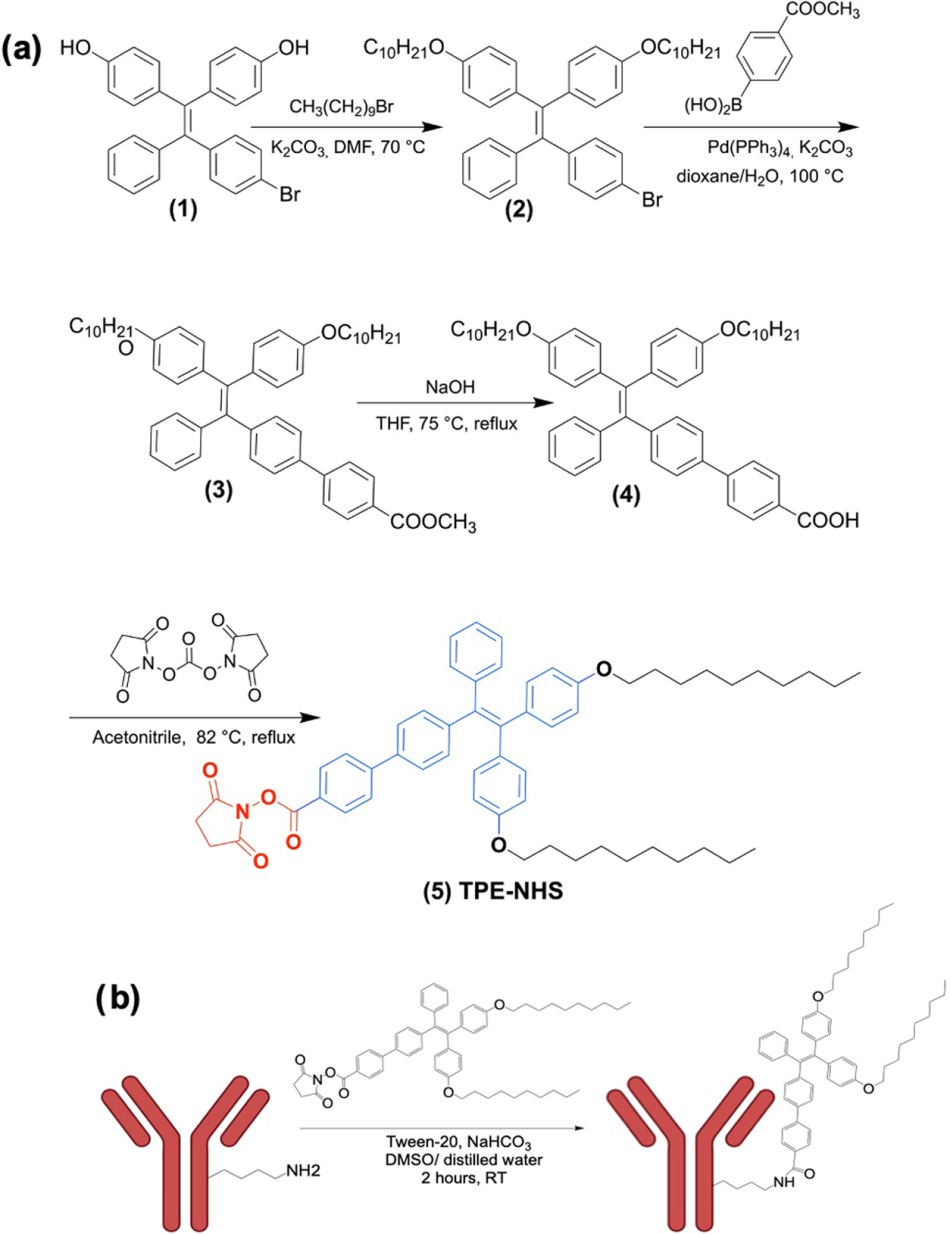
(a) Schematic of Synthesis of TPE-NHS (compound 5); the Three Key Components of TPE-NHS Are Indicated in Red (NHS Ester), Blue (AIE Group), and Black (Alkane Chain**)** and (b) Schematic of the AIE Sensor Development by Conjugation of TPE-NHS to the Lysin Residue of the Antibody

The spectral properties (absorption and emission) of TPE-NHS were characterized over a range of solvents with different polarities. According to the results in Figure S10, TPE-NHS demonstrates slight solvatochromic shifts in the absorbance and more significant shifts in the emission spectrum in DMSO, distilled water, and DMEM cell media. Previous work has shown that solvatochromism in organic molecules can be attributed to the stabilization of the electronic excited state of the molecule by the polar solvent, and this response is largely attributed to the hydrogen bonding abilities of the specific solvent.^44^ However, other solvent properties can also play a role. The observed results align with prior studies of solvatochromism in AIE molecules.^45−47^

Solubility testing confirmed that the amphiphilic TPE-NHS is soluble in mildly polar dimethyl sulfoxide (DMSO), which has a relative polarity of 0.44.^48^ The AIE response of TPE-NHS was confirmed by using two approaches. First, increasing the concentration of the molecule in the 99 (v/v) % solution of distilled water/DMSO results in the initiation of aggregation-induced fluorescence at concentrations above 10 μM (Figure S11a). Second, by increasing the relative volume ratio of distilled water/DMSO, the TPE-NHS begins aggregating, and the fluorescent emission in the system increases (Figure S11b).

The proposed application of this sensor is detecting the effect of Trastuzumab on HER2 clustering using the AIE response of TPE. Therefore, it is important to ensure that Trastuzumab does not directly interact with the fluorescent behavior of the TPE. A series of experiments were performed using the same range of Trastuzumab concentrations as in the cell line studies. No effect on the AIE response was observed (Figure S12). Additional details are in the SI.

### AIE Sensor Development and Characterization

To endow specificity to the TPE-NHS dye, we conjugated it to an antibody specific to the extracellular domain of the HER2. This conjugation process relied on NHS ester bonding with the amines that are part of the lysine residue, forming an amide bond with the HER2 antibody. Due to the potential of antibodies to denature and lose biological specificity, the NHS ester-amine conjugation chemistry is performed in aqueous solutions (Scheme 1b, Figure S13). To reduce the formation of aggregates and increase the bioconjugation yield, a micelle-mediated conjugation process using Tween 20 was performed.^49^ To optimize the Tween 20 concentration in the reaction solution, a series of TPE-NHS/HER2 antibody conjugation reactions using a range of Tween 20 concentrations were performed. Based on the results from the optimization study (Figure S14), 0.025 (v/v) % of Tween 20 was used in the antibody bioconjugation process.

To confirm the conjugation of TPE-NHS with the HER2 antibody, MALDI mass spectrometry was used. Results presented in Figure S15 show a simultaneous shift and broadening of the peak after the antibody conjugation process is performed. The software reported a 1.015 kDa shift in the *m*/*z* values of the HER2 antibody and TPE-HER2 Ab (TPE-NHS conjugated to HER2 Antibody) spectrum. Considering the fact that the expected molecular weight of each TPE-NHS post conjugation is 749 g/mol, this result demonstrates the conjugation of TPE to the HER2 antibody with an estimated average dye to antibody ratio of 1.35. Furthermore, a complementary SDS-PAGE assay further confirmed the formation of the TPE-HER2 Ab (Figure S16).

After conjugation, the optical absorption and emission wavelengths of the developed AIE sensor were characterized in 1× PBS. The absorption maximum of the conjugated molecule is 354 nm, and the emission wavelength is centered at 518 nm (Figure S17), which indicates a clear Stokes shift of 164 nm. Importantly, these results confirm that the fluorescent behavior of the dye has not been significantly altered by the bioconjugation process.

### Evaluation of the AIE Sensitivity, Cytotoxicity, and HER2 Target Specificity of the AIE Sensor (TPE-HER2 Ab)

**Figure 3:**
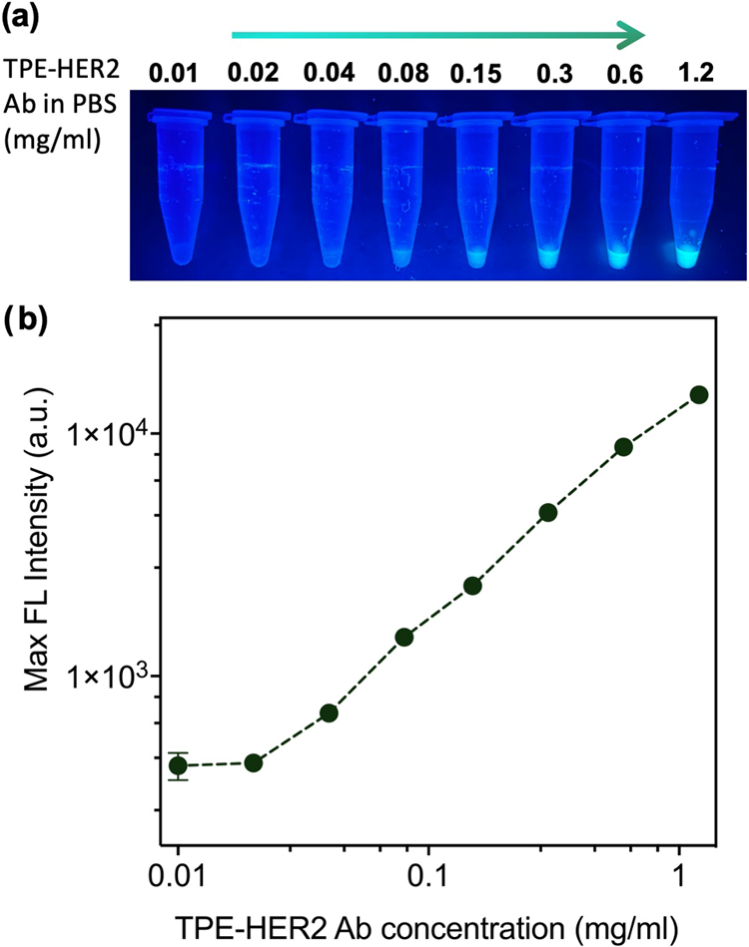
Fluorescent Behavior of TPE-HER2 Ab. (a) Optical images of different concentrations of TPE-HER2 Ab in 1× PBS excited by a 365 nm UV lamp. (b) Maximum FL intensity at each concentration of TPE-HER2 Ab in 1× PBS plotted against the concentration of TPE-HER2 Ab in each sample. In some cases, the error bars are not visible because they are smaller than the symbols.

The aggregation-induced emission behavior of TPE-HER2 Ab was analyzed by measuring the emission over a range of sample concentrations in 1× PBS (Figures 3a and S18). Results in Figure 3b show an increasing trend in the fluorescent emission intensity at concentrations above 0.04 mg/mL, which confirms that the expected AIE behavior of the AIE probe is maintained after antibody conjugation.

Before applying TPE-HER2 Ab in cell imaging applications, the cytotoxicity of the TPE-NHS probe was evaluated on two different breast cancer cell lines, SKBR3 and MCF7, using concentrations up to 50 μM. SKBR3 overexpresses HER2, and MCF7 is a HER2- low expressing cell line.^50^ The HER2 expression levels of these cell lines were confirmed using indirect immunofluorescent staining and Western blotting (Figure S19). Based on the CellTiter-Glo (CTG) assay results, no significant impact on cell viability was observed over the entire range of TPE-NHS concentrations studied as compared to the positive and negative controls (Figure S20). This observation confirms the low cytotoxicity of the compound and its potential applicability in live cell imaging studies.

In order for the TPE-HER2 Ab to accurately monitor the HER2 clustering process, it must selectively bind to HER2. The specificity of the TPE-HER2 Ab is evaluated by performing a colocalization, competition fluorescent imaging measurement using the SKBR3 cell line. Texas Red was selected as the second fluorophore because the excitation (emission) wavelengths of Texas Red do not overlap with the excitation (emission) wavelengths of TPE, allowing for colocalization to be easily determined by merging the fluorescent images (Figure S22). Both fluorophores were conjugated to the same type of HER2 antibody, removing the variability of the antibody binding site and affinity from the measurement. An identical series of colocalization imaging measurements is performed using Fluorescein in place of the TPE.

**Figure 4:**
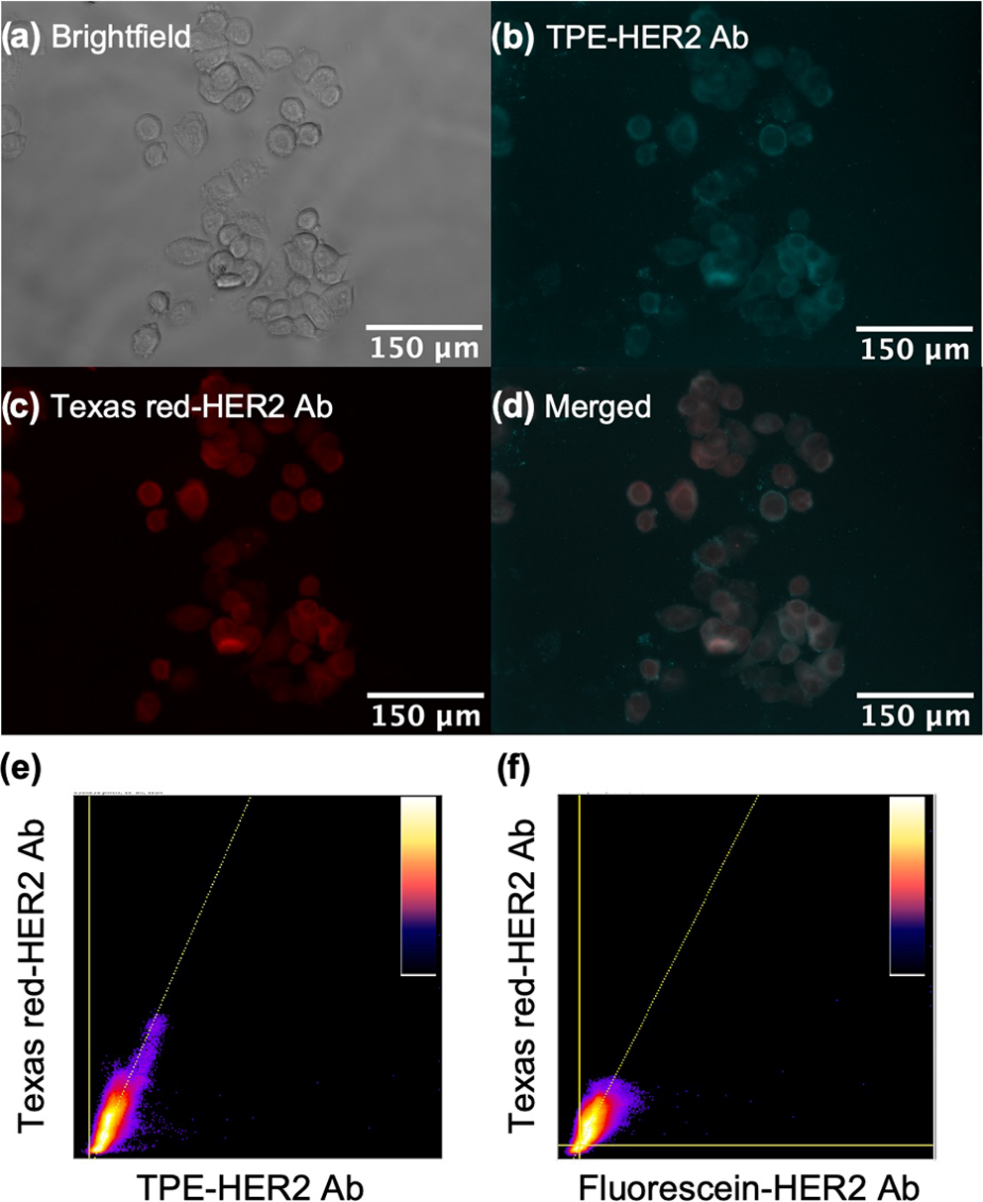
Colocalization competition analysis of TPE-HER2 Ab and Texas Red-HER2 Ab in SKBR3 (HER2-overexpressing) cells. Images of SKBR3 cells stained with 20 μg/mL of TPE-HER2 Ab and 20 μg/mL of Texas Red-HER2 Ab are shown in (a) bright-field channel, (b) TPE fluorescent channel, (c) Texas Red fluorescent channel, and (d) merged channel of TPE and Texas Red fluorescent channels. 2D intensity histogram of (e) TPE: Texas Red channels and (f) Fluorescein: Texas Red channels were used for photon intensity correlation analysis.

Bright-field and fluorescent images of the HER2-overexpressing SKBR3 cells labeled with Texas Red-HER2 Ab and TPE-HER2 Ab are shown in Figure 4a–c. The emission signal from each fluorophore is easily observable. One approach to quantify the signal quality is the signal-to-noise ratio (SNR). According to basic signal processing theory, an SNR above 1 is considered detectable. For the present images, the SNR of TPE and Texas Red are 10.23 ± 0.50 and 20.66 ± 2.22, respectively. Therefore, both fluorophores provide robust detection signals. The details of the SNR calculation are included in the SI.

When the Texas Red and TPE fluorescent images are merged (Figure 4d), the colocalization of the TPE and the Texas Red is qualitatively evident. The same colocalization experiment using Fluorescein-HER2 Ab and Texas Red-HER2 Ab was also performed as a control measurement, and similar results were obtained (Figure S23).

To quantitatively analyze colocalization, the photon intensities in the Texas Red and the TPE channels are spatially correlated (Figure 4e). This analysis is also performed for the previously discussed Fluorescein control measurement (Figure 4f). Furthermore, the Pearson correlation coefficient (PCC) of the TPE/Texas Red-HER2 Ab and the Fluorescein/Texas Red-HER2 Ab were calculated.^51^ Across multiple images, the PCC values of the TPE/Texas Red and the Fluorescein/Texas Red channels were calculated to be 0.74 ± 0.06 and 0.65 ± 0.03, respectively (Figure S24). Therefore, the TPE-HER2 Ab was able to specifically target HER2 on SKBR3 cells, setting the stage for HER2 cluster detection and analysis using this AIE-based imaging probe.

### AIE Sensor (TPE-HER2 Ab) for Detection of HER2 Clustering in Cancer Cells

The TPE-HER2 Ab imaging agent was used to detect HER2-HER2 interactions and HER2 clustering in two different breast cancer cell lines (SKBR3 and MCF7).

**Figure 5:**
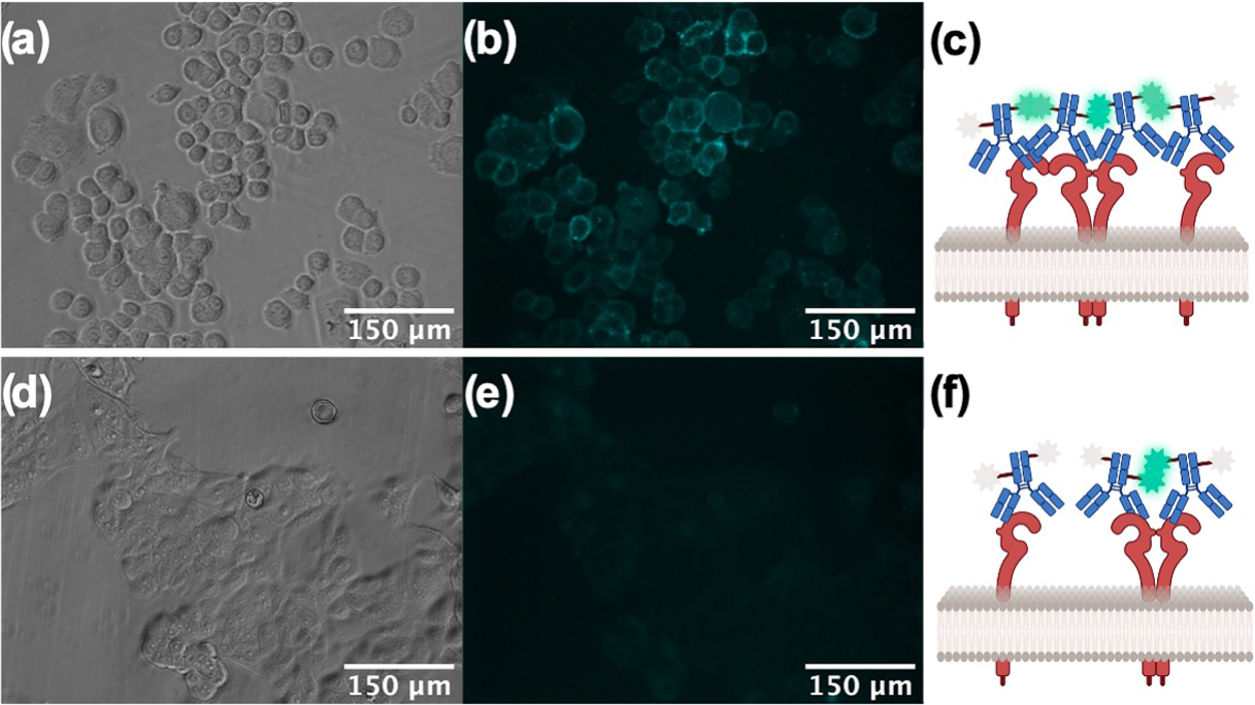
Bright-field and fluorescent images of (a, b) SKBR3 (HER2 overexpressing) and (d, e) MCF7 (HER2-low expressing) cells stained with 10 μg/mL of TPE-HER2 Ab along with the illustration of the TPE-mediated fluorescent turn-on process on the surface of (c) SKBR3 and (f) MCF7 cells.

One challenge with antibody-based assays is batch-to-batch variations in the antibody reactivity. To remove this variable and potential measurement confound, all measurements were performed with the same antibody lot.^52^As shown in Figure 5a–c, the fluorescent emission signal from the TPE in the HER2-overexpressing SKBR3 cells was clearly identifiable with an SNR of 9.67 ± 1.48. This signal is directly related to the AIE fluorescence mechanism of the TPE moiety. Namely, as the HER2 clusters form, the TPE molecular motion becomes restricted and the fluorescent intensity increases. In contrast, in the HER2-low expressing MCF7 cells, the fluorescent signal is barely detectable, with an SNR of 6.13 ± 0.76. (Figure 5d–f). This difference is due to a lack of HER2 clustering as well as a low HER2 concentration, and it is in agreement with the cell line validation assays.

Taken together, these observations in the SKBR3 and MCF7 cell lines confirm the ability of the TPE-HER2 Ab imaging agent to selectively target HER2 in cells and to detect HER2 clusters in the cell membrane. These findings set the stage for monitoring the dynamic process of HER2 cluster formation and disruption.

### AIE Sensor for Analyzing the Impact of Cancer Cell Therapeutic Treatment on HER2 Clusters

Trastuzumab (Herceptin) is a humanized monoclonal antibody that has been approved by the Food and Drug Administration (FDA) for patients with HER2-positive invasive breast cancer.^53^ This therapeutic is a HER2-specific antibody that binds the juxtamembrane portion of the HER2 extracellular domain.^54,55^ Several mechanisms of action have been proposed, including prevention of HER2-receptor dimerization.^54,56,57^ However, it is not clear if dimerization inhibition alone is sufficient to achieve the therapeutic results that are observed.^54,58^ Another hypothesis is that changing the biophysical pattern and distribution of HER2 clusters on the cell membrane gives rise to the observed effect.^55,59^ The TPE-HER2 Ab imaging agent developed here is uniquely suited to provide insight into this scientific question.

SKBR3 cells were seeded in a 96-well plate in triplicate and were treated with Trastuzumab concentrations ranging from 0 to 100 μg/mL for 2, 8, and 24 h. The concentration and time ranges were based on prior Trastuzumab studies.^59−61^ Subsequently, the TPE-HER2 Ab imaging agent was added to the plates following the developed direct immunofluorescence staining protocol. In parallel, the Fluorescein-HER2 Ab was used as a control. Fluorescent imaging was performed using a widefield fluorescent microscope with a 20× objective, and the images were analyzed and quantified using an in-house developed computational method. Details are included in the SI (Figure S25).

**Figure 6:**
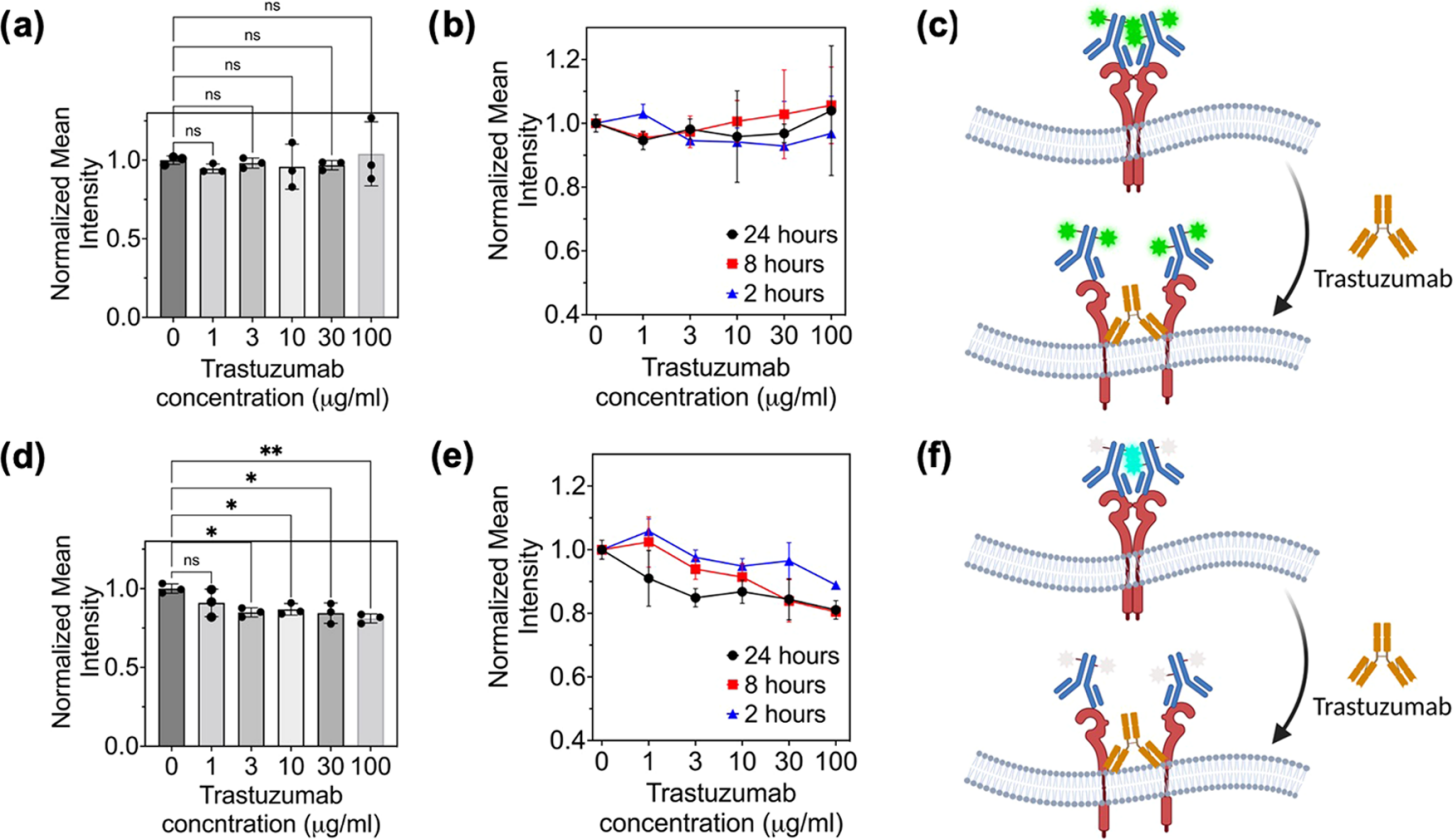
Normalized mean fluorescent intensity of SKBR3 cells stained with (a, b) 10 μg/mL Fluorescein-HER2 Ab and (d, e) 10 μg/mL TPE-HER2 Ab after treatment with a range of concentrations of Trastuzumab (0 μg/mL to 100 μg/mL) for (a, d) 24 h and (b, e) different time intervals of 2, 8, and 24 h all in one plot. The data is collected in triplicate, and on average, each data point consists of 30 SKBR3 cells. (*p < 0.05 and **p < 0.01). Schematic of HER2-overexpressing SKBR3 cell membrane indicating the fluorescent response of (c) Fluorescein-HER2 Ab and (f) TPE-HER2 Ab stained HER2 proteins after Trastuzumab treatment.

In the assay using Fluorescein-HER2 Ab, the average fluorescent intensity does not noticeably change in any of the Trastuzumab incubation times or concentrations used (Figures 6a–c and S26). Specifically, the SNR varies from 15.90 ± 0.90 to 15.74 ± 1.80 over the course of the 24 h imaging measurement, which is within the error of the SNR values. Therefore, even with a robust SNR value, there is no detectable change. Because Fluorescein-HER2 Ab is not sensitive to the proximity of the receptors, this result indicates that the HER2 expression is constant, and the absolute concentration of HER2 in the cell membrane is not changed. This result is expected. However, it does not provide insight into the mechanism of action of Trastuzumab and its impact on the HER2 clustering process.

In contrast, by monitoring the fluorescence intensity, TPE-HER2 Ab provides information about the HER2 clustering behavior. With 2 and 8 h of Trastuzumab treatment, a decrease in cluster formation is observed only at the highest concentrations (Figures 6e and S26). However, with 24 h of treatment, the clustering process is reduced with all concentrations above 1 μg/mL studied in this work (Figure 6d–e). In comparing the SNR values over the course of the 24 h imaging measurement, the SNR changes from 11.85 ± 0.55 to 10.73 ± 0.57.

This response to Trastuzumab is in agreement with prior studies exploring the impact of Trastuzumab on HER2 clusters in HER2-overexpressing cells.^58^ This result reveals that the decreasing fluorescent trend is due to the interaction between Trastuzumab and the HER2 clusters. These findings confirm the capability of our AIE sensor (TPE-HER2 Ab) for the detection and visualization of HER2 clustering dynamics upon exposure to an external stimulus like therapeutics.

## Conclusions

In summary, we developed a targeted imaging agent based on the combination of an AIE fluorophore with a monoclonal antibody and used it to monitor the clustering process of HER2 in response to a HER2-targeting therapeutic (Trastuzumab). The TPE- HER2 Ab conjugate system is specific to the target and can visualize the HER2 clustering process in HER2-overexpressing cells.^62^ Furthermore, using the developed fluorescent probe, the impact of Trastuzumab on the proximity of HER2 in the SKBR3 cells can be readily visualized and determined. The results demonstrate an easily generalizable method for studying the distribution and nanoscale localization of membrane-bound proteins. Given the target specificity of TPE-HER2 Ab and biocompatibility of TPE-NHS, this AIE sensor has the potential of being optimized for studying the dynamics of HER2 clusterization in live cells. Furthermore, while the focus of this work is on using HER2 antibodies, by shifting to peptide or similar engineered targeting moieties, quantification of the signal could be possible. This additional capability could lead to applications in developing improved therapeutics or in understanding membrane dynamics.^63,64^

## Supporting Information Available

DFT modeling of TPE-NHS, detailed synthesis procedures and characterization, UV–vis absorption and fluorescence spectra of TPE-NHS, effect of Trastuzumab on TPE-NHS emission, bioconjugation optimization imaging, MALDI and SDS-PAGE bioconjugation characterization, UV–vis absorption and fluorescence spectra of TPE-HER2 Ab, cell culture procedures, cell line validation through immunofluorescent imaging and Western blot, cell viability assay, SNR calculation protocol description, control multichannel fluorescent imaging, control colocalization assay, Pearson correlation coefficient data, detailed image analysis protocol description, and data for the AIE-based trastuzumab treatment assay (PDF)

## Abbreviations

AIE: aggregation-induced emission
TPE: tetraphenylethelene
HER2: human epidermal growth factor receptor 2
DMSO: dimethyl sulfoxide
DMEM: Dulbecco’s modified Eagle’s medium
BSA: bovine serum albumin
Ab: antibody
PBS: phosphate-buffered saline
BP: band-pass
NMR: nuclear magnetic resonance
MALDI: matrix-assisted laser desorption/ionization
SDS-PAGE: sodium dodecyl-sulfate polyacrylamide gel electrophoresis
CTG: CellTiter-Glo
SNR: signal-to-noise ratio
PCC: Pearson correlation coefficient
FDA: Food and Drug Administration
